# Complete Genome Sequence of Streptococcus ratti JH145

**DOI:** 10.1128/MRA.00144-20

**Published:** 2020-03-05

**Authors:** Sandra C. Garrett, Sara Olson, Brenton R. Graveley

**Affiliations:** a Department of Genetics and Genome Sciences, Institute for Systems Genomics, UConn Health, Farmington, Connecticut, USA; University of Rochester School of Medicine and Dentistry

## Abstract

We report here the complete genome sequence of Streptococcus ratti strain JH145. Streptococcus ratti is a cariogenic species of mutans streptococcus that has been isolated from rat and human teeth. The strain JH145, derived from strain BHT-2, is interesting for oral health because it does not produce cariogenic lactic acid but shows robust biofilm production.

## ANNOUNCEMENT

Streptococcus ratti is a species of mutans streptococcus that has been isolated from both rat and human teeth. Streptococcus ratti is closely related to Streptococcus mutans (1) and, like S. mutans, it has been isolated from carious lesions in humans and can cause experimental caries in rodent models (2–4). Strain JH145 was isolated from the parent strain BHT-2 following mutagenesis (5, 6); BHT-2 is a spontaneous streptomycin-resistant derivative of BHT (7). Although S. ratti BHT was isolated from human dental caries and was shown to be cariogenic (2), the mutant strain JH145 did not produce lactic acid or cause caries in a rodent model (6). To provide a genetic baseline for understanding tooth colonization and the formation of caries, we sequenced the complete genome of S. ratti JH145. To date, a complete genome sequence for S. ratti has not been cataloged in GenBank.

Streptococcus ratti JH145 was obtained from the ATCC (deposit number 31377) and grown in Todd Hewitt broth under microaerobic conditions (candle jar with ∼5% CO_2_ [8]). Genomic DNA was isolated using the Qiagen DNeasy PowerBiofilm kit (Qiagen, Valencia, CA). Illumina platform libraries were prepared using the NEBNext Ultra II library kit (New England Biolabs, Ipswich, MA). Libraries were sequenced on a MiSeq instrument (Illumina, San Diego, CA) with a 240 × 60-bp paired-end protocol, followed by fastq generation and adapter trimming with the generateFASTQ module within the MiSeq Reporter analysis package provided with the instrument. Following adapter trimming, the observed mean read lengths were 228 bases and 60 bases, and the mean *Q* scores were 37 and 36 for reads 1 and 2, respectively. In addition, long-read Oxford Nanopore libraries were prepared using the Ligation sequencing kit (Oxford Nanopore Technologies, Oxford, UK) and sequenced on a MinION R9.4.1 flow cell for 24 h. After sequencing, nanopore reads were base called using Guppy version 3.1.5 (9) with *Q* score filtering set to a quality threshold of 7. Porechop version 0.2.4 with default settings was used to trim adapter sequences (https://github.com/rrwick/Porechop). In total, 2,169,276 MiSeq reads and 602,986 Oxford Nanopore reads were generated. The mean read length for Oxford Nanopore reads was 4.9 kb. *De novo* assembly was carried out using SPAdes version 3.9.0 with BayesHammer error correction and default assembly settings (10). A single contig with 170× coverage was returned, and this was annotated using the NCBI Prokaryotic Genome Annotation Pipeline version 4.9 (11). To check that this contig represented the full, closed genome, we created a relinearized reference genome from it. In this reference genome, the first 10 kb of sequence was moved from the beginning of the contig to the end so that the original ends of the assembled contig now presented a continuous, closed sequence. The MiSeq short reads were then aligned to the relinearized reference genome with Bowtie 2 version 2.3.5.1 using default settings (12). Overall, 99.29% of the short reads aligned. In addition, Oxford Nanopore long reads with a *Q* score greater than 10 were aligned with minimap2 using default settings (13). Both alignment files were used to generate custom genome coverage tracks (14, 15) which were examined manually on the UCSC genome browser (16). Tracks showed even, uninterrupted coverage over the reference, including over the closed linearization point, which supports the conclusion that the genome is closed.

The complete S. ratti JH145 genome is 2,096,943 bp long with a GC content of 40.9%. A total of 1,941 protein coding genes was predicted (51 pseudogenes), along with 86 RNA genes and two CRISPR arrays. The CRISPR arrays were annotated as a type II-A and a type I-E system and contain 11 and 8 spacers, respectively. Unexpectedly, spacer content of the type II-A CRISPR array was identical to that found in S. mutans NCTC10920 (GenBank assembly number GCA_900638045), suggesting a close phylogenetic relationship (17). We used Mashtree (18) with default settings to analyze genome distances among S. ratti JH145, S. ratti FA-1 (type strain, genome in six contigs; GCA_000286075), and the following three strains of S. mutans: S. mutans UA159 (type strain; GCF_000007465.2), S. mutans GS-5 (GCA_000271865), and S. mutans NCTC10920 (GCA_900638045). JH145 showed greatest similarity to S. mutans NCTC10920, followed by S. ratti FA-1. Streptococcus mutans NCTC10920 is reported as S. mutans on GenBank but elsewhere is identified as S. ratti, a designation supported by our comparison. Our complete assembly for S. ratti JH145 will provide a resource for comparative genomics and dental health studies.

### Data availability.

The complete genome sequence of Streptococcus ratti JH145 has been deposited in GenBank under accession number CP043405 (assembly number GCA_008803015) and BioProject identifier PRJNA573065; raw reads were deposited in the Sequence Read Archive under accession numbers SRX6958171 (MiSeq) and SRX6958172 (Oxford Nanopore). Raw output files for the Oxford Nanopore sequencing (fast5 format) were also deposited under accession number SRR10977074.
